# Experimental evolution of UV resistance in a phage

**DOI:** 10.7717/peerj.5190

**Published:** 2018-07-09

**Authors:** Eric F. Tom, Ian J. Molineux, Matthew L. Paff, James J. Bull

**Affiliations:** 1 Department of Integrative Biology, University of Texas, Austin, TX, USA; 2 Department of Molecular Biosciences, University of Texas, Austin, TX, USA

**Keywords:** Adaptation, Mutagenesis, Genomics, Irradiation, Selection, Models

## Abstract

The dsDNA bacteriophage T7 was subjected to 30 cycles of lethal ultraviolet light (UV) exposure to select increased resistance to UV. The exposure effected a 0.9999 kill of the ancestral population, and survival of the ending population was nearly 50-fold improved. At the end point, a 2.1 kb deletion of early genes and three substitutions in structural-genes were the only changes observed at high frequency throughout the 40 kb genome; no changes were observed in genes affecting DNA metabolism. The deletion accounted for only a two-fold improvement in survival. One possible explanation of its benefit is that it represents an error catastrophe, whereby the genome experiences a reduced mutation rate. The mechanism of benefit provided by the three structural-gene mutations remains unknown. The results offer some hope of artificially evolving greater protection against sunlight damage in applications of phage therapy to plants, but the response of T7 is weak compared to that observed in bacteria selected to resist ionizing radiation. Because of the weak response, mathematical analysis of the selection process was performed to determine how the protocol might have been modified to achieve a greater response, but the greatest protection may well come from evolving phages to bind materials that block the UV.

## Introduction

Viruses are notorious for rapid evolution in response to new challenges, such as drugs, host immunity, and host range (Richman, 1996; Wasik & Turner, 2013; Longdon et al., 2014). Furthermore, the phenotypic magnitude of response can be profound. The speed and magnitude of viral evolution is so impressive that viruses, especially bacterial viruses (phages) have been a mainstay of experimental evolution studies for decades (Levin, 1988; Bull & Molineux, 2008; Doore & Fane, 2016). Yet viruses cannot adapt indefinitely, and an outstanding question is whether we can identify ‘evolution-proof’ antiviral agents to which viruses cannot adapt.

One cause of phage death that is likely of general importance in the natural environment of many phages is ultraviolet light (UV). UV is known to cause DNA damage that is both mutagenic and lethal, and it can also alter some peptide bonds. It is a major cause of phage mortality in marine environments, at least near the surface (Regan et al., 1992; Suttle & Chen, 1992) and presumably in many other environments that are exposed to sunlight. From an applied perspective, UV-induced phage death appears to be a major hurdle to the widespread use of phages to limit bacterial infections of crops (Jones et al., 2007, 2012; Iriarte et al., 2007). This specific application of plant phage therapy motivates understanding whether arbitrary phages can evolve meaningful levels of resistance to UV, but a bigger question is whether we can predict the evolution of such a response.

We thus sought to discover whether and how much a well-studied, laboratory phage might evolve resistance to UV killing. Given the limited genomic capacity of most phages to repair DNA, combined with the limited shielding of their genomes by their capsids, it may well be that improved UV survival is not possible. Any outcome is relevant to the general question of the limits of adaptation, as well as being of practical importance to the enterprise of agricultural phage therapy and any other use of phages in environments exposed to sunlight.

## The Nature of Survival and Selection in the Empirical System

The experimental protocol consisted of (i) exposing a lysate of phage to UV long enough to inflict a 4-log kill of the initial phage, then (ii) adding the survivors to a culture of cells and growing to culture lysis. The cycle was repeated 30 times, always using the same exposure duration. At the start, the phage population size before UV exposure was 10^7^–10^8^ (in 0.1 mL of lysate, the volume that was propagated), killed down by 10^4^, before grown up again, but because a constant duration of exposure was used, the extent of population reduction would have diminished as beneficial mutations evolved.

The dynamics of phage numbers in this design are conveniently partitioned into a lethal phase (with a survival rate of 10^−4^) and a growth phase, the decrease in density during the lethal phase being fully offset by the increase during the growth phase so that numbers remain constant from one cycle to the next. Our interest is in evolution of increased survival during the UV exposure so that, say, the same exposure that kills to 10^−4^ in the initial population kills to 10^−3^ or less after the evolution. Adaptation can happen in either phase, but we neglect any selection and adaptation that lies outside of the lethal phase.

There are three mathematical properties relevant to the effect of protocol on selection and evolution. We explain these as separate models, considered next.

### Model 1. Faster evolutionary progress is achieved with greater killing—once resistance mutations are present

An idealized survival function of a non-growing viral population exposed to UV may be assumed to follow an exponential decay with time: *S* = e^−*wt*^ being the survival probability after *t* min of exposure (*w* > 0). Smaller values of *w* have higher survival rates, with *w* = 0 experiencing no death. Letting *w* represent the survival rate of the wild-type, μ the survival rate of a beneficial resistant mutant (hence μ < *w*), the mutant has a relative survival advantage of

(1) $${\Omega _t} = {{{{\rm{e}}^{ - \mu t}}} \over {{{\rm{e}}^{ - wt}}}} = {{\rm{e}}^{(w - \mu )t}},$$

increasing without bound over time. The longer the duration of exposure, *t*, the greater the relative survival advantage of the mutant. The expression in (1) is a ratio of the two (positive) fitnesses, and with the constraint μ < *w*, the ratio is bounded to lie between 1 and +*∞*, a value of 1 indicating equivalent survival of the two types. This expression is for deterministic changes in abundance, as if both wild-type and mutant are so numerous that stochastic effects can be ignored.

If at the outset, the ratio of mutant abundance to wild-type abundance is ${{{n_\mu }} \over {{n_w}}}$, the ratio of abundance after one exposure of duration *T* will be

(2) $${{{n_\mu }} \over {{n_w}}}{{\rm{e}}^{(w - \mu )T}}.$$

Thus longer exposures will lead to greater increases in mutant frequency per cycle, so fewer cycles of longer exposure will be required to raise the mutant frequency to a desired level.

For our design of a 4-log wild-type kill over each of 30 cycles and with no adaptation to better survival, the cumulative killing would be 10^−120^. (As each cycle of 10^−4^ kill was followed by amplification, the cumulative kill is the overall measure from 30 cycles and is not a measure of kill applied in a single episode.) For a mutant whose survival after exposure is double that of wild-type [Ω_*T*_ = 2], its cumulative kill would be 10^−120^ × 2^30^. From (2), and given an initial frequency of 10^−5^, the mutant would reach a frequency (proportion of the population) of 0.9999 if originating at cycle 1, reach a frequency of 0.91 if first arising immediately before cycle 11, and reach a frequency of 0.01 if first arising immediately before cycle 21. A mere doubling of survival would be difficult to detect in many assays, but the cumulative effect of several such mutations would be easier to demonstrate. If needed, a longer exposure could be used to assay viability than is used in the selection, increasing the quantitative impact of the survival difference in the assay.

### Model 2. Greater killing risks extinguishing pre-existing mutants

The foregoing results assume large populations of both mutant and wild-type. Mutant abundance will often be low initially, so a long, lethal exposure risks killing all pre-existing beneficial mutants. If *N*_μ_ is the initial number of mutants before exposure (in the relevant volume), a lethal exposure of length *T* will leave an average of

(3) $${N_\mu }{{\rm{e}}^{ - \mu T}}$$

surviving mutants, independently of other genotypes in the culture. If the value of (3) is less than 1, there will be an average of less than one mutant remaining after exposure. For example, a mutant with fitness equivalent to wild-type (*w*), would survive the lethal phase (on average) only if it existed in at least 10,000 copies before exposure. With a 10-fold advantage over wild-type, it would need to be present in 1,000 copies. If no individuals with a particular mutation survive exposure, then that mutation will need to re-generate before it can possibly evolve to high frequency, and there will be less time for its ascent after it is re-introduced.

Any beneficial mutant that persists through the exposure bottleneck—or happens to be present at the end of the exposure phase because that is when it was generated—will then be amplified during the growth phase so that its numbers increase at least enough (on average) to survive the next cycle of killing. (This argument neglects any possible disadvantage during the growth phase.) So once a beneficial mutation survives the first exposure phase, it should progressively tend to escape extinction in subsequent cycles. The problem is achieving a sufficient initial abundance of the mutant so that it survives its first lethal exposure bottleneck.

One simple solution to the mutant extinction problem is merely to increase the pre-exposure population size. If mutant frequency remains unaltered with increasing population size and the same kill (duration of exposure) is maintained, then a larger initial population will allow more mutant individuals to survive the killing. The same effect is achieved by transferring a larger volume of the exposed pool (e.g. as if we had transferred 1 mL instead of 0.1 mL). This approach will work up to the point that protocol constraints allow the entire surviving population to be carried forward into the next cycle; if the surviving population is so large that it must be thinned to accommodate the limitations of equipment, the benefit of increasing pre-exposure population size may disappear.

### Model 3. Mutations generated by the lethal agent

The preceding models have assumed mutations are pre-existing. UV is mutagenic and so may generate mutations that improve resistance to UV. As UV also has lethal effects, longer exposures mean fewer total survivors but more beneficial mutations per survivor. Letting β be the beneficial mutation rate coefficient during exposure, the number of surviving individuals in a starting population of size *N* that acquire a beneficial mutation approximately follows

(4) $$N{{\rm{e}}^{-wt}}{{\rm\beta }}t$$

(for small β*t*; this approximation assumes that the new mutation does not convey a survival advantage in the generation in which it arises and that the lethal effects of exposure are independent of beneficial mutations). This formula reflects both an increased appearance of beneficial mutations with longer exposures (β*t*) as well as a decreased survival rate from longer exposures (e^−*wt*^). This quantity is maximized over *t* at *wt* = 1, hence an exposure that kills 63% of the population, or a survival of 0.37—a low level of killing (Mundry & Gierer, 1958; Gierer & Mundry, 1958; Bull, 2008). Thus, independently of the beneficial mutation rate, the highest success rate in acquiring a surviving beneficial mutation is with a relatively low kill rate.

In many protocols, a practical problem stemming from such mild killing is that it generates weak selection: e^(*w* − μ)*t*^ in (1) is very near 1.0. For example, the per cycle level of killing used here (10^−4^) was more than three orders of magnitude higher than 0.37 (10^−0.434^). Thirty cycles of a 10^−4^ survival is a kill equivalent to 276 cycles of a 0.37 survival per cycle. Although a survival fraction of 10^−4^ may be harsh on pre-existing mutants, conducting the equivalent total selection for a survival rate of 0.37 becomes tedious without automation. A more relevant question is then whether the number of surviving beneficial mutants generated by UV in (4) is expected to exceed 1 after exposure. If even a few beneficial mutants persist, then a high killing reaps the benefits of rapid evolution without extinguishing the mutational input needed for evolution.

Expression (4) may be interpreted as a product: the number of survivors times the genomic beneficial mutation rate. In our study, the first factor approached 10^4^ in each cycle, so if the genomic beneficial mutation rate was 10^−5^ or higher, then the first 10 cycles of exposure would be expected to generate at least a few such mutations in the survivors. We do not know the beneficial mutation rate for any length of exposure, but there are indicators of the T7 genomic mutation rate under some mutagenic conditions. For T7 grown in a high concentration of the mutagen nitrosoguanidine (not lethal to the free phage), approximately four viable mutations are produced per genome per cell infection (Springman et al., 2010). For the mutagen hydroxylamine used to kill down to a survival between 10^−3^–10^−4^, the genomic mutation rate is up to eight mutations per genome (Bull et al., 2013). The T7 genome has 4 × 10^4^ nucleotide sites, so if a mutation at even one site was beneficial, a rate of four mutations per genome would generate a genome with a beneficial mutation at least once in 10^4^ genomes (this calculation neglects mutational biases and it further combines different types of mutations at a single nucleotide site). The fitness effects of individual random mutations have been estimated in phages, with 13 of 45 randomized point mutations being measured as beneficial in the DNA phage ϕX174 (although the effects of most of the 13 were not significantly different from 0 (Domingo-Calap, Cuevas & Sanjuán, 2009)). How closely these calculations apply to UV mutagenesis is unknown, but they suggest that the protocol may well have introduced enough mutations to allow adaptive evolution—if mutations with a benefit exist.

### Integrating the models

The three models apply to different aspects of the protocol. ‘Model 1’ describes the magnitude of selection as a function of exposure. The model applies once mutants have risen to an abundance allowing them to escape stochastic loss and also applies to protocols in which several different phage types are grown to high numbers and mixed before exposure/selection. ‘Model 2’ is merely a deterministic description of the number of individuals expected to survive an exposure, given an initial abundance and fitness. Since the initial number of mutations will often be unknown, its purpose is primarily qualitative—a caution against the overuse of selection and to highlight the possibility than a highly lethal exposure may kill all rare mutants. Model 3 provides insight to the relationship between kill and the introduction of beneficial mutations, specifically when the lethal agent is also the source of new mutations. The optimal exposure has the general property of being independent of the actual beneficial mutation rate. If the optimal exposure is applied successively to each cycle, then Model 3 accrues to the generation of mutations within each cycle, whereas Models 1 and 2 accrue to the selection and survival of mutants generated in previous cycles.

The calculations in these models were developed after the experimental selection was completed. The design had been chosen to impose strong selection, and the original intent was to limit the study to 10 cycles—for which any detectable progress would require strong selection. However, upon analysis at 11 cycles, it was decided to extend the study ∼20 additional cycles. A 30 cycle experiment would have afforded a less intense selection and greater propensity for beneficial mutation accumulation. Nonetheless, UV resistance was obtained under the current design, indicating that at least some beneficial mutations survived the UV-induced bottlenecks.

## Methods

### Strains

IJ1133 [*Escherichia coli* K-12, F^−^ Δ*lacX74 thi* Δ(*mcrC- mrr*)*102*::Tn*10*] was the host used for phage growth and non-selective plating. IJ512K [K-12 Δ*lacX*74 *supE*44 *galK*2 *galT*22 *mcrA rfbD*1 *mcrB*1 *hsdS*3/F′ *lac* Kan-R] was the F-bearing host used to selectively plate phage carrying T3 gene *1.2*. IJ1126 [K-12 *recB*21 *recC*22 *sbcA*5 *endA gal thi Su*^+^ Δ(*mcrC*- *mrr*)*102::Tn10*] was the host used for transfection of T7 DNA (Heineman, Bull & Molineux, 2009). Phage Δ*16* and complementing plasmids were reported elsewhere (Chang, Kemp & Molineux, 2010).

An isolate of presumed wild-type T7 (T7^+^) was used to initiate the study. This phage was later found to carry two mutations differing from the ‘standard’ wild-type (Genbank V01146.1): 15094 G→T and 29258 A→G. A variant of T7^+^ (JB14-37) was created by recombination over plasmid pMK-RQ-T7trxAdspB to insert T3 gene *1.2* into and largely replacing T7 non-essential gene *3.8* (Schmerer et al., 2014). In contrast to T7^+^, JB14-37 is able to plate on IJ512K.

Recombinant genomes were created by restriction digestion of genomes with *Bst*EII, which cuts T7 at wild-type base 20066. Other enzymes whose sites were limited to either the left or right fragment were used in conjunction with *Bst*EII, so that only one of the two halves remained intact. Digests were precipitated and, without gel purification, cognate fragments were ligated and transfected into IJ1126 and plated.

### Selection protocol

A single phage line was subjected to 30 cycles of exposure, each cycle followed by non-selective amplification. Host IJ1133 was used for amplification and most platings; for amplification, it was grown at 37 °C in minimal M9 glucose (0.2%) to approximately 10^8^ cells/mL, phage were added and the culture grown to lysis. The phage concentration in the lysate was typically 10^8^–10^9^ per mL. About 5 mL of lysate were then added to a polystyrene Petri dish and exposed to 302 nm UV for 120 s on the surface of a UVP® transilluminator (bulbs UVP 34-0042-01). For the initial phage, this exposure led to an approximately 4-log decline in viable phage, but the exposure was fixed to 120 s throughout regardless of possible evolutionary increases in survival. 0.1 mL of the survivors were added to a new culture of IJ1133, and the process repeated.

### Fitness estimation

The value of Ω in (1) gives a relative survival benefit of one phage over another. (We omit time notation, assuming that all exposures are for the same length of time—2 m in this study.) We want to know its value; here Ω was estimated in two ways. Motivated by the definition $\Omega = {{{S_{\rm{\mu }}}} \over {{S_{w}}}}$ (where *S* is survival, subscripted according to wild-type or mutant), one method simply calculated a ratio of observed survivals of each phage exposed separately of the other. The estimator of Ω replaces the true values with the estimated values (all estimated values are indicated with ^):

$$\hat \Omega = {{{{\hat S}_\mu }} \over {{{\hat S}_{w}}}}.$$

*Ŝ* for a phage type is merely the ratio of phage density observed after exposure to phage density observed before exposure.

The second estimator relied on relative survival in a mixed stock of phage. To use this estimator, proportions of each type of phage are determined before and after UV exposure. Letting *N* be the initial density of the phage mix (with wild-type phage at a frequency of 1 − *p* in the mix) and *K* be the post-exposure density of the phage mix (with wild-type at a frequency of 1 − *p*′), the ratio

(5) $${{Kp'/K({\rm{1}}-p')} \over {Np/N({\rm{1}}-p)}}$$

is simply the ratio of numbers of each type of phage in the mix before and after exposure. Except for sampling error,

(6) $${{Kp'} \over {Np}} = {{\rm{e}}^{-{\rm{\mu}}}},$$

(7) $${{K({\rm{1}}-p')} \over {N({\rm{1}}-p)}} = {{\rm{e}}^{-w}},$$

so that the ratio in (5) is the empirical manifestation of

(8) $${{{{\rm{e}}^{-{\rm{\mu }}}}} \over {{{\rm{e}}^{-w}}}} = \Omega .$$

As (5) reduces to

(9) $${{p'/({\rm{1}}-p')} \over {p/({\rm{1}}-p)}},$$

Ω may be estimated without knowing *N* or *K*, merely from the proportions of each type of phage in the post-exposure sample and the proportions of each type of phage in the pre-exposure sample. In contrast to the first method of Ω estimation, here there is no need to know the absolute phage densities in either sample, only the proportions of each type. This method can be used to measure fitness of a single mutant genome compared to wild-type in the same sample but can also be extended to compare two genomes in different mixes, each compared to the same standard; in the latter case, the ratio of the two Ω values gives the Ω between the two genomes.

### Statistics

Two tests of statistical significance were employed. One simply measured the phage titre for each of two types (*X*, *Y*) before and after UV exposure, (subscripts *a* and *b* indicating after and before, respectively). The ratios *X_a_*/*X_b_* and *Y_a_*/*Y_b_* are the respective survivals of each phage type, and if the survival rate of type *X* is the same as of type *Y*, the ratio

(10) $${{{{{X_a}} /{{X_b}}}} \over {{{{Y_a}} /{{Y_b}}}}} = 1,$$

hence

(11) $${\rm{log}}({X_a}) - {\rm{log}}({X_b}) + {\rm{log}}({Y_b}) - {\rm{log}}({Y_a}) = 0.$$

With multiple, independent measures of the different survivals the logged values will be approximately normally distributed, and the left side of (11) provides a statistic that will follow a *t*-distribution with degrees of freedom equal to 4 less than the number of survival measures.

The preceding *t*-statistics require multiple, independent samples. An alternative approach to testing sampling distributions is to do so empirically, essentially by ‘bootstrapping.’ Thus, the distribution of phage type *X* in a sample of size *N* in which the observed number of type *X* is *O_X_* may be simulated by drawing *N* independent, uniform random variables each with probability *O_X_*/*N*. Any statistic that uses *O_X_* may be reconstituted with the simulated numbers to obtain its distribution.

### Genome sequences

Population sequences from cycles 11 and 30 were generated by MiSeq at the University of Texas GSAF. Average read depth was ∼4500X at cycle 11, ∼3000X at cycle 30. Fastq sequence files were analysed with breseq (Deatherage & Barrick, 2014); some statistics were obtained from a C program written to sort breseq output. Mutations whose frequencies were less that 0.001 were excluded from analysis, to reduce possible attribution of sequence error to mutation. Sequence of the initial T7 was considered to be wild-type (GenBank V01146.1) except possibly for the four high-frequency changes observed at cycle 11. The status of those mutations in the initial T7 was evaluated by Sanger sequences (or size) of PCR products over the relevant regions; two of the four were observed in the ancestor, as given in Table 1 below. Fastq files are deposited in the NCBI Sequence Read Archive (SRP142350).

**Table 1:** High frequency mutations observed.

Bases	Genome change	Protein change	Frequency
1,084–3,212	Deletion	Loss of *0.4*–*0.7*	1.0*
32,855	T→C	I754T (*16*)	0.89
33,491	C→T	A966V (*16*)	1.0
35,095	A→G	N158D (*17*)	1.0*

**Notes:**

This table includes all changes above a frequency of 15% in cycles 11 or 30. Those listed are from cycle 30; mutations seen in cycle 11 are a subset of those seen at 30 and are indicated with a superscript ^*^ in the last column (both were at frequency of 1.0 in cycle 11). Two mutations differing from the published wild-type sequence were present in the ancestor used to start the adaptation: 15094 G→T (A248S, gp5) and 29258 A→G (K312E, gp15).

## Results

### UV resistance evolves

**Figure 1:**
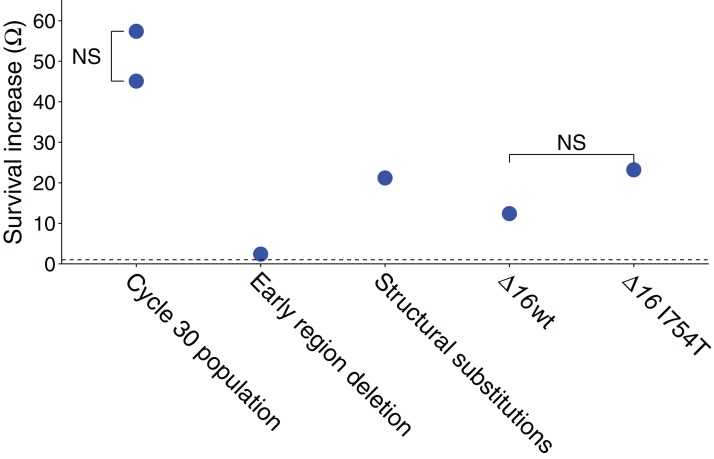
Ω values represent the survival advantage of the phage indicated over wild-type. All illustrated values are explained in the text, and the statistics of all values are also given in the text (each value shown is significantly greater than 1.0 by at least *P* ≤ 0.001). ‘NS’ indicates the absence of a statistically significant difference between the two samples compared. The dashed line represents Ω = 1, the value at which the two phages have equal survivals. The ‘early region deletion’ and ‘structural substitutions’ separately test the cycle-30-evolved 2.1 kb deletion from the three substitutions evolved in genes *16* and *17*. The two Δ*16* columns are from the test in Table 2.

A single phage line was subjected to 30 cycles of UV exposure; survival improved significantly. The improved survival of the evolved phage (cycle 30) over the starting phage (Ω) was estimated in two ways, one comparing separate measures of absolute survivals between the initial and evolved phages, the other a relative measure using the change in proportion of the two phages in a mixed stock (see Methods; as all exposures were 2 m, the time subscript on Ω is omitted). Comparing absolute survivals, the evolved population exhibited a Ω = 45.1-fold improvement in survival (Fig. 1; this test was based on five independently exposed samples of wild-type and five of evolved virus, *t*(8) = 10.5, *P* < 0.001; survival data were converted to log_10_ to estimate means and to conduct the statistical test; the mean log_10_ difference was 1.65 *±* 0.11).

Absolute survival calculations were based on platings of each stock, both before and after exposure. The plaque forming units are affected by several sources of error—intrinsic Poisson variation in phage per volume, variation in pipette volume, and chance effects in plaque initiation per phage that affects plating efficiency. A relative measure of change in survival was therefore also used to independently estimate the survival difference. This assay used a mix of the evolved phage and a derivative of a wild-type phage carrying a selectable marker (JB14-37; see Methods). The mixed sample was exposed to UV for a single cycle. Exposed and unexposed samples were separately plated on a permissive host and individual plaques then stabbed onto the restrictive host, yielding proportions of the evolved phage before and after irradiation. The change in proportion of the evolved phage gives its survival relative to the other phage in the mix. By this method, the relative advantage of the evolved line over wild-type was Ω = 57.4 (Fig. 1), significantly larger than 1 but compatible with the absolute fitness superiority estimate of 45.1: using the bootstrap test, the sample yielding Ω = 57.4 was found to generate a value of Ω = 45.1 or less in 25% of trials but a value of 1.0 or less in none of 2 × 10^5^ trials. (This assay was based on a single stock irradiated once for the treatment and not irradiated for the control, with a total of over 450 plaques assayed from each of the irradiated and non-irradiated populations.)

### Population sequences reveal a few candidate resistance mutations

The population of T7 was sequenced after 11 and 30 cycles (Table 1). Two differences from the ancestor were at high frequency by cycle 11: a 2.1 kb deletion of the early region, as is typical in other adaptations of T7 (Studier et al., 1979; Cunningham et al., 1997), and a point mutation in gene *17* (35,095 A→G, N→D in codon 158). By cycle 30, those two mutations plus two additional high frequency changes were found, the latter both affecting gene *16*. The deletion was an in-frame fusion of the first 53 codons of gene *0.3* with gene *1*, encoding the RNA polymerase gene, but the RNA polymerase gene was truncated for its first 14 codons. T7 RNA polymerase is essential for phage growth, so transcription and other functions must have been retained in this fusion.

At cycle 11, many uncommon mutations were observed (mutations whose population frequencies were <5%). These mutations were found at nearly 11,000 sites in the ∼40 kb genome, and although individually rare, they were collectively so abundant—occurred at so many sites—that the average individual genome carried approximately 49. The dominant mutations were GC→TA transversions (80%), in contrast to expectation from repair of cyclobutane pyrimidine dimers or pyrimidine:pyrimidone 6,4, photoproducts (Karran & Brem, 2016; Schuch et al., 2017), or the domination by GC→AT mutations observed following nitrosoguanidine or hydroxylamine mutagenesis of T7 (Springman et al., 2010; Bull et al., 2013). Sequences from cycle 30 revealed a much lower level of uncommon mutations per genome (approximately 11), and the mutation spectrum was no longer dominated by GC→TA.

Because of the discrepancy between cycles 11 and 30, the rare mutations were characterized more fully. The modest difference in read depth between cycles 11 and 30 was one possible concern, so these new analyses forced a read depth of at least 1,500 per site. When requiring each mutation to have a minimum frequency of 0.001, the average mutation load per genome was 40.9 vs 6.0 (for cycle 11 and 30, respectively). Using a minimum mutation frequency of 0.005 (or 0.01), the corresponding numbers were 16.8 vs 1.1 (1.3 vs 0.8). Thus the overall load of mutations was from the low-frequency spectrum, chiefly frequencies less than 0.01, and the large difference between cycles 11 and 30 was not due to differences in the coverage.

The improved survival of the adapted populations is plausibly attributed to the high-frequency mutations, but the effect sizes may differ greatly. Recombinant genomes between the initial and evolved (cycle 30) populations were generated by reciprocal exchanges of *Bst*EII fragments (which cuts the genome at 20066), separating the left-end deletion from the right end substitutions in *16* and *17*; mutational status of the recombinants was verified by sequences of PCR fragments. UV sensitivity of recombinant isolates exhibited a significant Ω = 2.4 benefit of the ‘left end’ of the evolved genome, carrying the deletion, and a significant Ω = 21.2 benefit of the right side, carrying the gene *16* and *17* mutations (Fig. 1; for both tests, *P* < 10^−4^ that Ω ≤ 1 by bootstrap using the combined data). The product of benefit from both halves is approximately as expected for the total. As noted under Model 1, above, a mutant with a relative survival benefit of even two would be expected to rise to high frequency by cycle 30 but be barely detectable by cycle 11. As the deletion was virtually fixed by cycle 11, its advantage in UV protection is either higher than 2 in the presence of the *17* mutation (i.e. epistasis), or it has an advantage in the growth phase (as seen with an early-region deletion in another study using M9 media Springman et al., 2012).

We note that recombinants are clonal (isolated as plaques) and may thus carry some low-frequency mutations from the evolved population at cycle 30 (mutation status was confirmed for the high-frequency mutations by size or sequence of PCR products from the relevant regions but those methods would not have detected mutations in other parts of the genome). Any deleterious fitness effects of uncommon mutations were likely minor, as the phage population was subjected to several generations of liquid growth to generate a phage stock for DNA preparation; this growth phase would have purged strongly deleterious mutations.

### Does the mechanism of genome entry explain the benefit?

At first glance, none of the changes makes sense for UV protection. For gp17 (tail fibre) it is not obvious how the mutation could improve survival—unless UV specifically destroyed the wild-type protein and thereby ravaged adsorption capacity. Indeed, UV (300–400 nm) fluxes have actually been shown to inactivate T7 by causing an adsorption defect (Hartman & Einsenstark, 1982). Alternatively, our experimental regime may have allowed adaptation of a tail fibre that then binds a UV-shielding agent such as cellular debris, but as the observed substitution lies within or near the tail-binding, rather than the cell-binding, domain this possibility seems unlikely.

One of the changes in gene *16* is plausibly protective, but only indirectly. The internal virion protein (gp16) has multiple functions, a critical one being in catalysing genome internalization (García & Molineux, 1996; Kemp, Gupta & Molineux, 2004; Chang, Kemp & Molineux, 2010). The wild-type phage genome enters the cell in three phases. The initial ∼1 kb enters by a proton motive force-dependent mechanism that involves both T7 gp16 and gp15 present in the mature virion (Chang, Kemp & Molineux, 2010); this process internalizes the three early major promoters for *E. coli* RNAP. Host-mediated transcription from these promoters then pulls the following ∼6 kb into the cell. T7 RNAP is encoded on this 6 kb and when synthesized internalizes the remainder of the genome via transcription from T7-specific promoters.

It is well established that *cis–syn* cyclobutane pyrimidine dimers, pyrimidine:pyrimidone 6,4, photoproducts, and other UV-induced DNA damage block transcription by *E. coli* and T7 RNAP when the lesions are present on the transcribed strand (Sonohara, Iwai & Kuraoka, 2015). However, the lesions have little to no effect when present on the non-template strand. Thus half of UV-induced DNA damage would be expected to interfere with or completely inhibit T7 genome internalization.

The biochemical mechanism of T7 gp15/gp16-dependent genome internalization is incompletely understood, although there is no evidence that it is strand or DNA sequence-dependent. However, the gp16-I754T mutation found in UV-resistant T7 has been shown to allow internalization of the entire genome in the absence of any transcription (García & Molineux, 1996; Struthers-Schlinke et al., 2000; Kemp, Gupta & Molineux, 2004). Thus the potential exists for a phage harboring gp16-I754T to completely internalize a UV-damaged genome where cellular processes might repair damage. The possible magnitude of any such benefit in the evolved phage is limited, of course, as the evolved phage survives over wild-type only by approximately 50 genomes in every 10,000 that die from UV (and over half of this effect is attributable to a deletion in the early region).

To test whether I754T might convey a large benefit, we took advantage of a T7 gene *16* deletion mutant (Δ*16*), which can be grown on hosts containing complementing plasmids (Chang, Kemp & Molineux, 2010). By growing Δ*16* on a host that provides wild-type *16*, or alternatively, on a host that provides the gp-I754T substitution, it is possible to generate T7 Δ*16* phage differing only in the structural gp16. Each stock was mixed with a wild-type competitor carrying a T3 *1.2* insert, the same wild-type competitor used in the experiments described above (JB14-37).

The relative changes in Δ*16* frequency within each stock before and after UV exposure gives a survival measure of Δ*16* relative to the competitor, and a comparison between the Δ*16* grown on wild-type vs I754T gives the benefit of I754T. The data reveal a benefit of the Δ*16* genome grown on either plasmid, with only a two-fold greater survival of being assembled with gp16-I754T over assembly with wild-type gp16 (Fig. 1; Table 2). Although both $\hat \Omega $ values are significantly greater than 1.0 (*P* < 10^−4^ by bootstrap tests), the difference in $\hat \Omega $ values is not significant (*P* ∼ 0.5 by the bootstrap test), nor does the two-fold extra benefit of gp16-I754T over gp16-wt begin to approach the 21-fold benefit observed for the right-end portion of the evolved genome. Thus, the gp16-I754T substitution does not appear to explain the survival benefit of the evolved phage.

**Table 2:** Relative survival tests.

	Δ*16*/*16*^+^		T7^+^/*16*^+^
	*16wt*	*16* I754T	
Before UV	66/34	45/55	39/61, 55/45
After UV	96/4	95/5	40/60, 47/53
$\hat \Omega $	12.4	23.2	1.04, 0.73

**Notes:**

Each term in a ratio is a number of plaques (out of 100) that typed a particular way. The denominator is always the number of plaques for the *16*^+^ phage (14–37), a wild-type T7 derivative that carries a T3 gene *1.2* insert in T7 gene *3.8*. The numerator is the number corresponding to the other phage in the mix, as given at the top of the column. Ω values apply to the phage in the numerator. The rightmost column evaluates the effect of the small insert in JB14-37 against a T7^+^ phage lacking the insert; there is no evidence of an effect.

The unexpected finding from this test is a large benefit—over 10-fold—of the *16* deletion when carrying a wild-type gp16 (Fig. 1; Table 2). By contrast, the early region deletion in the evolved phage was found to confer an approximately two-fold benefit. There is, of course, expected to be some benefit of the *16* deletion because it removes approximately 10% of the genome, and nearly all of the deleted region is essential—so the target size for UV-induced mutation is reduced. From a target-size effect alone, we would expect the survival benefit to be

(12) $${{{{\rm{e}}^{-0.9L}}} \over {{{\rm{e}}^{-L}}}} = {{\rm{e}}^{0.{\rm{1}}L}}.$$

For a wild-type survival of 10^−4^, *L* = 9.21, and e^0.921^ = 2.5 well short of the observed value of Ω = 12.4 (*P* ∼ 0.0002 by the bootstrap test). Thus, there appears to be an advantage of the 16 deletion beyond merely the reduction of genomic target size for UV, and the additional benefit of a phage carrying gp16 with I754T is not enough to explain the survival benefit of the ‘right’ end of the evolved phage. This test does raise the possibility of some interaction effect of *16*, however, as the overall benefit of the *16* deletion and I754T mutation is roughly the same as observed for the right end of the evolved phage. It may also be significant that the I754T mutation was one of two mutations in *16*, and it was not even fixed by cycle 30—consistent with it not having the largest effect. It is also noteworthy that the left-end deletion of (non-essential) 2.1 kb is expected to have a benefit of Ω = 1.6 from the reduction in target size alone; this value is significantly lower than that observed, further suggesting that the benefit of deletions stems from more than just a reduced target size (*P* < 0.03 by the bootstrap test; although statistically significant, such a difference is small relative to the other fitness effects to be accounted for).

## Discussion

The successful selection of UV resistance in phages is interesting from several perspectives. (1) A priori, radiation might seem to be an ‘evolution-proof’ lethal agent for a phage—that the DNA is unavoidably unprotected. However, phages that encode their own DNA metabolism genes would seem to at least have the opportunity to repair UV-damaged DNA, much as in bacteria (Alcántara-Díaz, Breña-Valle & Serment-Guerrero, 2004; Harris et al., 2009). (2) Phages have been used to treat bacterial infections of plants, but phage are subject to rapid killing in sunlight, due at least in part to UV exposure. If phages can be selected for UV resistance, their half-life in the field can be extended. (3) The adaptive response to UV irradiation appears to involve deletions of non-essential genes. Such an adaptive response may constitute a rare demonstration of an error catastrophe, an evolutionary phenomenon much discussed and commonly associated with viral quasispecies (see below).

The evolution here led to increased survival under UV exposure. The effect was an approximately 50-fold improvement over a 4-log killing of the ancestor. This improvement may or may not have a profound utility in an industrial setting. A 50-fold advantage in a 10^−4^ kill reduces the kill to 10^−2.3^. If the total kill of the ancestor was 10^−12^, the evolved strain would experience a kill of 10^−6.9^. There are many settings in which this level of kill might still be too great. But for any application in which phage were already being used, a 50-fold improvement would likely have a major impact.

For contrast, the directed evolution of *E. coli* to resist a mix of β and γ irradiation (decay of ^60^Co) led to a nearly four-orders of magnitude of improvement (Harris et al., 2009). When measured on a scale such that the kill of the initial population was between four and five logs, the kill of the evolved population was less than one log. Thus, on a survival scale similar to that of this study, the evolved improvement was approximately 100-fold greater in *E. coli* than here. Also in contrast to the present findings, the *E. coli* recovery was due to mutations affecting DNA repair genes.

A close parallel to our T7 study was conducted with *E. coli* K-12 (Goldman & Travisano, 2011). Several lines were subjected to 60 cycles of selection with a 25 s UV exposure that killed 0.75 of the parental generation (thus close to the optimum given by model 3). The lines improved three-fold over the ancestor. At first glance, the Ω = 3 value is less than that obtained here, but a comparison of values between the studies must be adjusted for the different kill levels per cycle. A kill to 10^−4^ survival (used here) is the same as 6.6 consecutive exposures that each have kill to 0.25. The three-fold improvement in the bacterial study translates into an improvement of 1,478 (3^6.6^ for a kill to 10^−4^), thus nearly 30-fold better than obtained with T7. This is of course to be expected as bacteria have many more potential modes of UV damage repair than phage. Furthermore, greater evolutionary progress was obtained with far less of a cumulative kill.

The T7 results present a few anomalies. First, instead of protection evolving via changes in DNA metabolism, the majority of protection was due to mutations in genes encoding essential structural proteins. Testing of one of those mutations in a deletion background failed to explain the effect and revealed a further anomalous magnitude of increase in resistance associated with the deletion.

A second puzzle is that the population at cycle 11 exhibited an abundance of rare mutations (frequencies mostly less than 1%), such that the average genome harbored nearly 50 of them. By cycle 30, the incidence was reduced to just over 11 per genome. The cycle 11 spectrum of rare mutations was chiefly GC→TA transversions. A large component of GC→TA has been seen with UVA irradiation, and both UVA and UVB generate 8-oxoG products that can lead to transversion mutations (for reviews see Pfeifer, You & Besaratinia, 2005; Karran & Brem, 2016; Schuch et al., 2017). Furthermore, although UVA is usually considered a longer wavelength range than the 302 nm exposure used here, there is no strict boundary between UVA and UVB. In addition, our protocol may have included factors that modified the realized wavelength, such as a brief warm-up period for the UV lamp and exposure of a crude lysate through polystyrene. The change in mutation abundance and spectrum between cycles 11 and 30 may divulge a mutation spectrum that was highly sensitive to unmeasured variables. The anomaly does raise the intriguing possibility that the observed mutation spectrum and abundance may be a useful diagnostic of nuances in the actual exposures. However, even if the mutation spectrum changed, a more striking result is that the realized mutation rate must also have changed, as indicated by the decline in mutation incidence. Indeed, had mutations stopped altogether by cycle 11, many of the existing mutations should have been maintained into cycle 30 at only somewhat reduced frequency—unless they were moderately to highly deleterious (a possibility compatible with the overwhelming majority being transversions). However, at this point, only speculation can be offered for these incongruities.

With the benefit of hindsight informed by the models, especially Models 2 and 3, evolutionary progress in our study may have benefitted from a less stringent kill level per cycle. A 10–100-fold lower kill per cycle, even though still much higher than the optimal kill of 0.63 from model 3, would likely have improved the retention of rare mutants. The very abundance of rare mutations at cycle 11 suggests that UV was in fact a major contributor to mutational input in our study (as supported by many previous studies on mutagenic effects of UV) and thus reinforces the relevance of model 3 in designing an optimal protocol. At the same time, that so many mutations were present leads us to expect that there was an abundance of mutations available for selection despite our stringent selection. Our protocol was driven by an intuitive understanding of increased selection from a large kill (Model 1), but such strong selection is ill advised until mutations are moderately abundant. This post hoc awareness of the different effects of the kill employed suggests the possible benefit of a hybrid protocol—use weak selection early until mutations start to ascend, then increase selection in later phases. It may also be possible to benefit from other hybrid protocols, such as splitting the culture and exposing different subcultures to different kill levels before combining them for the next exposure. We have not formally investigated the mathematics of hybrid protocols and merely offer these few suggestions to inspire a formal search for improved methods.

Despite the present success in evolving partial resistance to UV exposure, a substantially greater phage tolerance to UV may require a combination of measures rather than merely longer selection of the naked phage. One approach is to suspend phages in protective media before application (Born et al., 2015). In addition to merely adding protective media, however artificial selection may also be used to improve phage survival specifically in such contexts. The mathematical considerations provided here that describe selection apply equally to naked phages as to phages that are exposed in complex environments, provided those environments are maintained throughout the cycles of selection. In a different environment, the phage response may involve binding to protective agents instead of blocking the UV directly, and some blocking agents may provide far better protection than a naked phage could ever evolve. Indeed, the method of ‘phage display’ is one of evolving phages to bind substrates, and a phage display library using a randomized motif in the capsid protein in the presence of an appropriate agent may greatly accelerate the evolution of protection from UV. Likewise, if a variety of phages is available for an application, it will be straightforward to apply strong selection to identify the subset of phages best able to resist UV: when different genomes are being competed, all of the types will be common initially and thus not subject to extinction of rare types.

A deletion of the non-essential early region accounted for a small but significant two-fold improvement in survival. The deletion itself fused *0.3*—which encodes a phage defense against type I restriction-modification systems—in frame with the RNAP gene *1*, truncating a few of its 5′ codons. Although deletions of the early region are commonly observed in T7 grown in rich media (Studier et al., 1979; Cunningham et al., 1997), the benefit in those cases is from improved growth; here the benefit measured was explicitly improved virion survival or plating efficiency. (There may also have been a benefit during the growth phase, but such an advantage would not have appeared in the survival assay.)

Enhanced resistance to UV was observed for the two deletions tested here, a deletion of the early region that evolved during the adaptation (mentioned in the preceding paragraph), and a deletion of gene *16* that was used in an assay. In both cases, the benefits of the deletions must come from the effect on the genome itself or its effect on intracellular life cycle rather than from an effect on the protein components of the virion: the deletion of *16* was complemented with a plasmid, so the virion proteins were unaffected, and none of the early gene products missing from the early-region deletion occur in the virion. There are several plausible mechanisms by which a deletion could enhance survival. Most prominently, deletions reduce target size for lethal mutations. This possibility is obvious for the *16* deletion (*16* is essential) but may also apply to the deletion of non-essential early genes, if UV-induced mutations in their DNA could be lethal—as with unrepairable lesions that could not be transcribed or replicated. Reducing the target size for lethal UV damage would thus increase survival. In both cases, however, the proportional reduction in genome size did not explain the full magnitude of benefit, so some additional mechanism must be invoked. But to the extent that a reduced target size explains some of the advantage, this latter model is one form of an evolutionary phenomenon known as an error catastrophe, normally difficult to demonstrate (Eigen, 2002; Bull, Meyers & Lachmann, 2005). Error catastrophes represent the evolution of a genome (or genome network) robust to the effects of deleterious mutation. In this case, the error catastrophe would be a reduced genomic lethal mutation rate and thus improved survival in the face of higher mutation, even though the deletion might be deleterious in the absence of UV.

The benefit of a deletion could possibly stem from other mechanisms instead—and thus not represent an error catastrophe. Higher virion survival might result from the reduced DNA density in the phage head, although how a reduced density would specifically enhance UV protection is not clear. Finally, and relevant only to the early-region deletion, some of the deleted genes interfere with host function (e.g. *0.7* shuts off host transcription). If host functions are important to repairing the damaged phage genome, the loss of those interfering phage activities could improve cell-mediated repair. There are no doubt other mechanisms that might explain the survival benefit of deletions without invoking a reduction of mutation target size (i.e. without invoking an error catastrophe). Discriminating among alternatives may be feasible with specific manipulations of the phage. For example, introducing premature stop codons in genes of the early region would eliminate potential interference of those proteins on cell-mediated repair without deleting the DNA.

Our calculations of the effect of selection (Eq. 1) assume that the phage population is exposed uniformly to the lethal agent. If instead there are protected subpopulations (e.g. shielded by debris), then the killing of those subpopulations will be attenuated; in the extreme, they will escape the lethal agent entirely. Protected subpopulations will reduce the effect of selection, perhaps profoundly so. As an extreme case, a refuge fully protecting a mere 0.0001 fraction of a population exposed to an otherwise complete kill could give the impression of a 4-log kill yet would impart no selection at all. Analysis of the kill curve over a wide range of killing would suggest whether protected subpopulations are likely to be present. However, prolonged use of a protocol that enabled limited protection in refuges might select phages that were progressively better at becoming protected.

## Conclusions

Modest success was achieved in adapting phage T7 to resist killing by UV—a 40–50-fold improvement evolved in response to an exposure that initially killed to 10^−4^. The mutational basis of the adaptation was complex, including deletions as well as substitutions in structural proteins, but was puzzling in that no changes were observed in DNA metabolism genes. Further improvement might be attained with a longer duration of weaker episodes of selection and also by evolving the phage to bind a blocking agent. It will be interesting to compare the UV-sensitivities of different wild phages to discover whether they are approximately equally sensitive to UV, or perhaps that some are far more robust than others.

## Additional Information and Declarations

### Data Availability

The following information was supplied regarding data availability: Sequences are deposited in NCBI Sequence Read Archive (SRP142350).
